# Arylamino-*nor*-β-lapachone derivative-induced apoptosis in human prostate cancer cells: involvement of NAD(P)H:quinone oxidoreductase (NQO1)

**DOI:** 10.1186/1753-6561-7-S2-P12

**Published:** 2013-04-04

**Authors:** Bruno C Cavalcanti, Hemerson IF Magalhães, Felipe AR Rodrigues, Manoel O Moraes, Cláudia Pessoa

**Affiliations:** 1 Department of Physiology and Pharmacology, Federal University of Ceará, P.O. Box 3157, CEP 60430-270, Fortaleza, Brazil

## Background

β-lapachone, a DNA repair inhibitor, has been recognized as important prototype with activity against cancer cells devoided of cytotoxicity in non-tumor cells. NQO1 is a reductive enzyme that is important for the activation of many bioreductive quinones. Thus, differential levels of NQO1 in tissues, including tumors, can provide a target for an enzyme-directed approach to cancer therapy. Herein, we aimed to evaluate the role of NQO1 on the cytotoxicity of 3-arylamino-*nor*-β-lapachone derivative using the prostate DU-145 (NQO1-overexpressing) and LNCap (NQO1-deficient) cells.

## Materials and methods

Compound cytotoxicity was evaluated by the MTT assay, and apoptosis and free radicals were observed by flow cytometry. Also, comet assay was performed to evaluate the DNA strand breaks induced by quinonoid compound. For all experiments, cells were treated in the presence or absence of dicoumarol (NQO1 inhibitor).

## Results

The 3-arylamino-*nor*-β-lapachone derivative showed cytotoxic activity (IC_50_ 2.98 µM) after 24 h exposure. In order to determine the mechanisms involved in cytotoxicity, cells were treated with increasing concentrations (1, 2 and 4 µM) of compound during 4 h. After exposure, apoptosis signals, DNA damage and free radicals production were observed. Coadministration of dicoumarol (50 µM) abrogated 3-arylamino-β-lapachone derivative-mediated cytotoxicity and downstream apoptotic end points.

## Conclusions

In summary, NQO1 may be a pharmacologically exploitable target for therapy against certain tumors using lapachone compounds.

## Financial Support

CAPES, CNPq, BiotechCell, FUNCAP.

